# Interferon-γ Is a Crucial Activator of Early Host Immune Defense against *Mycobacterium ulcerans* Infection in Mice

**DOI:** 10.1371/journal.pntd.0004450

**Published:** 2016-02-10

**Authors:** Raphael Bieri, Miriam Bolz, Marie-Thérèse Ruf, Gerd Pluschke

**Affiliations:** 1 Swiss Tropical and Public Health Institute, Basel, Switzerland; 2 University of Basel, Basel, Switzerland; Fondation Raoul Follereau, FRANCE

## Abstract

Buruli ulcer (BU), caused by infection with *Mycobacterium ulcerans*, is a chronic necrotizing human skin disease associated with the production of the cytotoxic macrolide exotoxin mycolactone. Despite extensive research, the type of immune responses elicited against this pathogen and the effector functions conferring protection against BU are not yet fully understood. While histopathological analyses of advanced BU lesions have demonstrated a mainly extracellular localization of the toxin producing acid fast bacilli, there is growing evidence for an early intra-macrophage growth phase of *M*. *ulcerans*. This has led us to investigate whether interferon-γ might play an important role in containing *M*. *ulcerans* infections. In an experimental Buruli ulcer mouse model we found that interferon-γ is indeed a critical regulator of early host immune defense against *M*. *ulcerans* infections. Interferon-γ knockout mice displayed a faster progression of the infection compared to wild-type mice. This accelerated progression was reflected in faster and more extensive tissue necrosis and oedema formation, as well as in a significantly higher bacterial burden after five weeks of infection, indicating that mice lacking interferon-γ have a reduced capacity to kill intracellular bacilli during the early intra-macrophage growth phase of *M*. *ulcerans*. This data demonstrates a prominent role of interferon-γ in early defense against *M*. *ulcerans* infection and supports the view that concepts for vaccine development against tuberculosis may also be valid for BU.

## Author Summary

*Mycobacterium ulcerans* is the causative agent of Buruli ulcer (BU), a slow progressing ulcerative skin disease. The mode of transmission of *M*. *ulcerans* remains unknown and only little is known about the early stages of the disease and the nature of protective immune responses against this pathogen. Given the increasing evidence for an early intracellular growth phase of *M*. *ulcerans*, we aimed at evaluating the impact of cell-mediated immunity for immunological defense against *M*. *ulcerans* infections. By comparing wild-type and interferon-γ-deficient mice in a BU mouse model, we could demonstrate that interferon-γ is a critical regulator of early host immune defense against *M*. *ulcerans* infections, indicative for an important role of early intracellular multiplication of the pathogen. In mice lacking interferon-γ the bacterial burden increased faster, resulting in accelerated pathogenesis. The observed differences between the two mouse strains were most likely due to differences in the capacity of macrophages to kill intracellular bacilli during the early stages of infection.

## Introduction

Buruli ulcer (BU), caused by infection with *Mycobacterium ulcerans* (*M*. *ulcerans*), is a progressive disease of the skin and subcutaneous tissue. The disease is primarily affecting West African rural communities, but has also been reported from America, Australia and Asia. The pathogenesis of BU is mainly attributed to mycolactone, a macrolide exotoxin produced by *M*. *ulcerans* [1]. Mycolactone is essential for bacterial virulence and is highly cytotoxic for a wide range of mammalian cell types *in vitro* and *in vivo*, including fibroblasts, keratinocytes and adipocytes [1–4]. Injection of the toxin induces the formation of necrotic non-inflammatory lesions similar to BU lesions. In addition to the induction of apoptosis, mycolactone possesses immunosuppressive characteristics and has been demonstrated to downregulate local and systemic immune responses [5,6], by interfering with the activation of immune cells such as T-cells, dendritic cells, monocytes and macrophages [7–10]. Furthermore, exposure to mycolactone results in complete inhibition of tumor necrosis factor alpha (TNFα) production by monocytes and macrophages, affects T-cell homing and interferes with the expression of T-cell receptors as well as co-stimulatory molecules including CD40 and CD86 [6–12].

Despite these immunosuppressive features of mycolactone, sera of individuals living in BU endemic regions frequently contain *M*. *ulcerans*-specific antibodies, demonstrating that many individuals develop immune responses associated with exposure to *M*. *ulcerans* without developing clinical disease [13,14]. Moreover, high mRNA levels for the cytokines interferon-γ (IFNγ), interleukin-1β and TNF-α were found in human BU lesions, indicating that the innate immune system is activated at the site of infection [15]. Reports on spontaneous healing of BU [16,17], and a partial protective effect of Bacille Calmette-Guérin (BCG) vaccination in humans and experimentally infected mice [18–22] are all factors indicating that clearance of the *M*. *ulcerans* infection by the immune system is possible, in particular before large clusters of mycolactone producing extracellular bacteria have formed. These clusters are located in necrotic subcutaneous tissue of advanced BU lesions and are no longer reached by infiltrating leukocytes.

Antibodies against surface antigens of *M*. *ulcerans* do not seem to have a protective effect [23], indicating that cellular, and in particular type 1 helper (T_H_1) cell responses [1,24] are more important in immune defense against BU than humoral responses.

IFNγ is critical for host defense against intracellular pathogens. In *Mycobacterium tuberculosis* (*M*. *tuberculosis*) infections, IFNγ produced by T_H_1 cells, but also CD8 cytotoxic T (T_c_) cells and NK cells, renders the macrophage competent to kill intracellular bacteria by overcoming the pathogen-induced block in phagosome-lysosome fusion and by producing microbicidal effectors such as nitric oxide (NO), resulting in host cell apoptosis and clearance of the bacteria [25–28]. During *M*. *ulcerans* infection, an early intra-macrophage growth phase seems to play an important role before the formation of extracellular clusters of mycolactone producing bacteria can be observed [6,29–31]. Protection mediated by IFNγ stimulated macrophages seems to be impaired by the suppression of IFNγ production after local build-up of mycolactone [32].

Here we have re-evaluated the role of IFNγ for host immune defense against *M*. *ulcerans* by comparing progression of the infection in IFNγ knockout and wild-type mice experimentally challenged with a fully virulent *M*. *ulcerans* isolate.

## Methods

### Ethical statement

This study was carried out in strict accordance with the Rules and Regulations for the Protection of Animal Rights (Tierschutzgesetz SR455) of the Swiss Federal Food Safety and Veterinary Office. The protocol was granted ethical approval by the Veterinary Office of the county of Vaud, Switzerland (Authorization Number: 2657).

### Mouse procedures

Mice were kept in specific pathogen-free facilities at the Ecole Polytechnique Fédérale de Lausanne (EPFL), Switzerland. All experiments were performed under BSL-3 conditions either in 8 week old female C57Bl/6 wild-type mice or mice homozygous for the Ifng^tm1Ts^ targeted mutation (IFNγ^-/-^, B6.129S7-Ifng^tm1Ts^/J, Jackson Laboratory). In total, 20 wild-type and 20 IFNγ^-/-^ mice were infected and 5 animals per group were euthanized at week 1, 3, 5 and 8 and used for qPCR analysis (3 mice) or histopathology (2 mice). The experiment was performed in two independent biological replicates. Animals were infected with the *M*. *ulcerans* strain S1013 isolated in 2010 from the ulcerative lesion of a BU patient from Cameroon [33] which is regularly tested for the production of mycolactone by ASL extraction and subsequent cytotoxicity tests on L929 fibroblasts as well as for the presence of the virulence plasmid pMUM001 by PCR. The bacteria were cultivated from a low passage cell bank for six weeks in Bac/T medium (Biomerieux, 251011), pelleted by centrifugation and diluted in sterile PBS to a stock concentration of 125 mg/ml wet weight. Mice were infected subcutaneously into the hind left foot pad with 30 μl (about 1 x 10^4^ bacilli as determined by qPCR corresponding to 5 x 10^3^ CFUs when plated on 7H9 ager plates) of an appropriate dilution of the stock suspension in sterile PBS. Progression of the infection was followed by weekly measurements of the foot pad thickness using a caliper. At weeks 1, 3, 5 and 8, groups of mice were euthanized and pictures of the feet were taken using a compact camera (WG-20, RICOH). The foot pads were aseptically removed for determination of the bacterial load by quantitative real-time PCR (qPCR) or for histopathological analysis.

### Determination of bacterial load by qPCR

Feet designated for the quantification of *M*. *ulcerans* were cut into 4 pieces using a scalpel and transferred to hard tissue grinding tubes (MK28-R, Precellys, KT03961-1-008.2). Next, 750 μl sterile PBS was added and feet were homogenized using a Precellys 24-Dual tissue homogenizer (3 x 20 s at 5000 rpm with 30 s break). The lysates were transferred into new tubes and the remaining tissues were homogenized for a second time after adding 750 μl of sterile PBS. The two lysates were pooled and used for DNA isolation. The DNA was extracted from 100 μl of a 1/20 dilution of the foot pad lysates as described by Lavender and Fyfe [34] and the isolated DNA was analyzed for insertion sequence (IS) 2404 by qPCR as previously described [34]. The number of genomes per foot pad was calculated according to the standard curve established by Fyfe *et al*. [35].

### Histopathology

Mouse feet used for histopathological analysis were fixed in 10% neutral-buffered formalin solution (4% formaldehyde, Sigma, HT501128-4L) for 24 hours at room temperature, decalcified in 0.6 M EDTA and 0.25 M citric acid for 14 days at 37°C and transferred to 70% EtOH for storage. After dehydration and embedding in paraffin, 5 μm thin sections were cut. Sections were then deparaffinised, rehydrated, and stained with Haematoxylin/Eosin (HE, Sigma, 51275-500ML, J.T. Baker, 3874) or Ziehl-Neelsen/Methylene blue (ZN, Sigma, 21820-1L and 03978-250ML) to stain for mycobacteria according to WHO standard protocols [36]. Finally, the sections were mounted with Eukitt mounting medium (Fluka, 03989) and pictures were taken with an Aperio scanner or with a Leica DM2500B microscope.

### Western blot analysis

Ten μg of *M*. *ulcerans* whole cell lysate was resolved on a 1-well 4–12% gradient gel (NuPAGE Novex 4–12% Bis-Tris Gel, Invitrogen, NP0330BOX) using MES running buffer and transferred to nitrocellulose membranes with the iBlot dry-blotting system (Novex, Life Technologies) according to the manufacturer’s recommendations. The membrane was blocked in 5% skim milk / PBS overnight at 4°C, cut into thin strips and incubated with the indicated sera diluted 1:400 in 1% skim milk / PBS-Tween-20 for 1.5 hours. After washing in 1% skim milk / PBS-Tween-20, the membrane was incubated for 1 hour with HRP-conjugated goat anti-mouse IgG γ-chain secondary antibody (Southern Biotech, 1030–05) diluted 1:4000 in 1% skim milk / PBS-Tween-20. Blots were developed using the ECL Western Blotting Substrate (Pierce, 32106).

### Statistics

A non-parametric Mann-Whitney test (Prism GraphPad) was used for statistical analysis of foot pad thickness measurements. Because of the small sample size for each group at a certain time point, the measurements of the bacterial loads were analyzed using non-parametric regression models according to the Brunner-Langer method [37]. The factor of interest, increase in bacterial burden between week 3 and 5 in the case of (1) and bacterial burden at week 5 in the case of (2) was included in a model to determine its effect on the examined outcome. In (1), because all 4 time points are compared in a second model, results from the regressions were adjusted for multiple comparisons using Dunnett-Hsu’s correction. The global effect of group, time point and of the interaction of group by time point were first tested [38].

## Results

### *M*. *ulcerans* infections progress faster in mice lacking IFNγ

**Fig 1:**
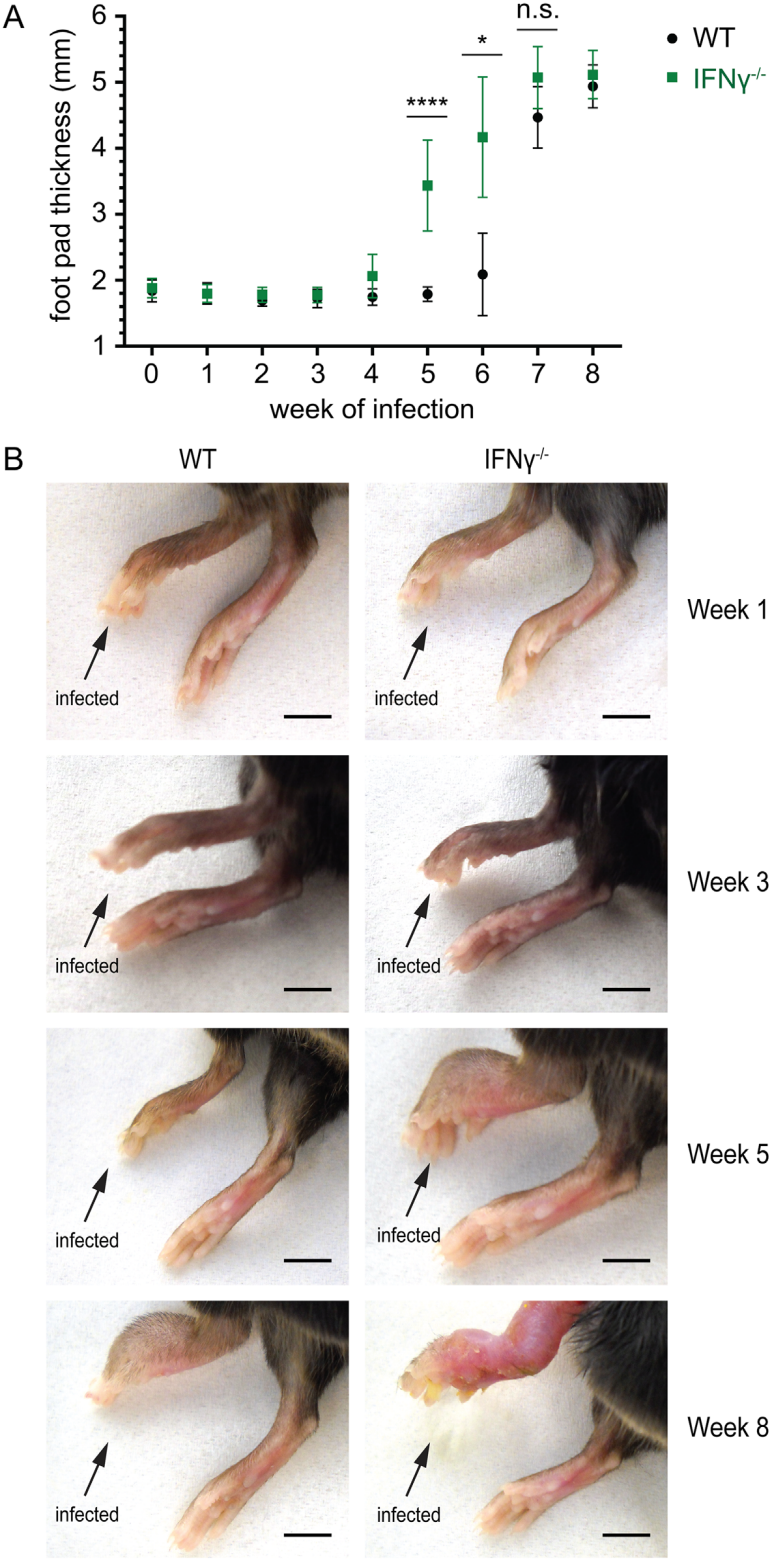
Faster progression of *M*. *ulcerans* infection in IFNγ-deficient mice. WT and IFNγ^-/-^ mice were infected into the left hind foot pad with *M*. *ulcerans* and the progression of the disease was followed by weekly measurements of the foot pad thickness (A) and documented with pictures of the infected feet (B). (A) IFNγ^-/-^ mice exhibited an accelerated progression of *M*. *ulcerans* infection. At weeks 5 and 6, the foot pad thickness was significantly higher in IFNγ^-/-^ mice than in WT animals. Mean values of the foot pad thickness (mm) are shown, the error bars represent the S.D. P values were calculated using non-parametric Mann-Whitney test. ****, P ≤ 0.0001; *, P ≤ 0.05; n.s., not significant. (B) Pictures of representative feet taken 1, 3, 5 and 8 weeks after infection. At week 5, all mice deficient for IFNγ^-/-^ had swollen feet. No swelling was observed in WT mice at this time point. Eight weeks after infection, foot pad swelling was observed for both WT and IFNγ^-/-^ mice but the macroscopic disease symptoms were more severe in mice lacking IFNγ. Scale bars represent 5 mm.

In order to evaluate the role of IFNγ in host immune defense against *M*. *ulcerans* infections, we infected 8 week old female C57Bl/6 wild-type (WT) mice and mice homozygous for the Ifng^tm1Ts^ targeted mutation (IFNγ^-/-^) into the left hind foot pad with 1 x 10^4^ *M*. *ulcerans* bacilli as determined by qPCR. Progression of the disease was followed by weekly measurements of the foot pad thickness with a caliper. While all of the ten IFNγ^-/-^ mice displayed strong swelling of the infected foot pads after 5 weeks of infection, no swelling was observed for the ten WT animals (Fig 1A). After 6 weeks of infection, one of the remaining five WT animals also started to show swelling of the infected feet. However, there was still a significant difference in foot pad thickness between the two different groups, which only resolved by week 8 (Fig 1A).

Complementary to the determination of the foot pad thickness, we documented the disease progression with pictures of the infected feet at 1, 3, 5 and 8 weeks after infection (Fig 1B). At week 5 infected foot pads of all ten WT mice did not show any macroscopic difference to the non-infected right control foot pads (Fig 1B). In contrast, all infected feet of the ten IFNγ^-/-^ mice were swollen and showed signs of inflammation (Fig 1B). Although the difference in the foot pad thickness resolved after 8 weeks of infection, the infected feet of the IFNγ^-/-^ animals were more inflamed and clearly more ravaged at this time point (Fig 1B).

### IFNγ-deficient mice display more extensive tissue necrosis and oedema formation than WT mice

**Fig 2:**
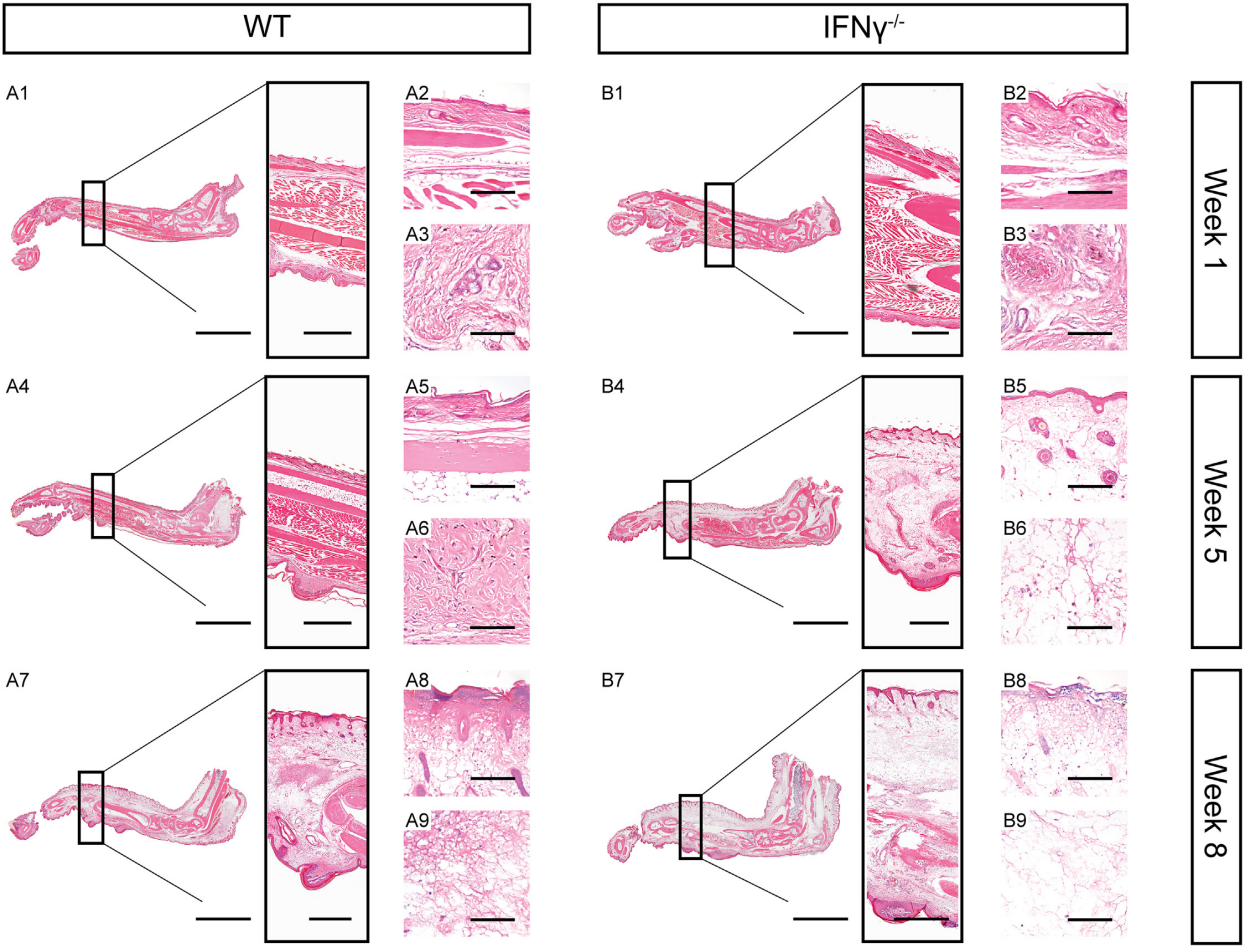
Extensive tissue necrosis and oedema formation in mice lacking IFNγ. HE stained histologic sections of foot pads from representative WT (A) and IFNγ^-/-^ (B) mice 1, 5 and 8 weeks after infection with *M*. *ulcerans*. Scale bars represent 5 mm (A1, A4, A7, B1, B4 and B7, left), 1 mm (A1, A4, A7, B1, B4 and B7, box), 150 μm (A2, A5, A8, B2, B5 and B8) and 80 μm (A3, A6, A9, B3, B6 and B9).

Histopathological analysis of two representative foot pads was performed to evaluate whether the increased foot pad thickness in IFNγ^-/-^ mice at week 5 was caused by cellular infiltration or mainly by oedema formation. While no changes in tissue integrity were observed after 1 week of infection in both groups (Fig 2A1–2A3 and 2B1–2B3), the two IFNγ^-/-^ mice displayed massive oedema formation and tissue necrosis after 5 weeks of infection (Fig 2B5, 2B4 and 2B6, respectively). Both are typical hallmarks of BU pathogenesis [39,40]. In contrast, the foot pads of the two WT animals were devoid of oedema formation or tissue necrosis at this time point (Fig 2A4–2A6). Eight weeks after infection the foot pads of the IFNγ^-/-^ mice were still more oedematous and necrotic than those of the WT animals and the infection even affected the adjacent joints and legs (Fig 2A7–2A9 and 2B7–2B9).

### Enhanced bacterial multiplication in IFNγ-deficient mice

**Fig 3:**
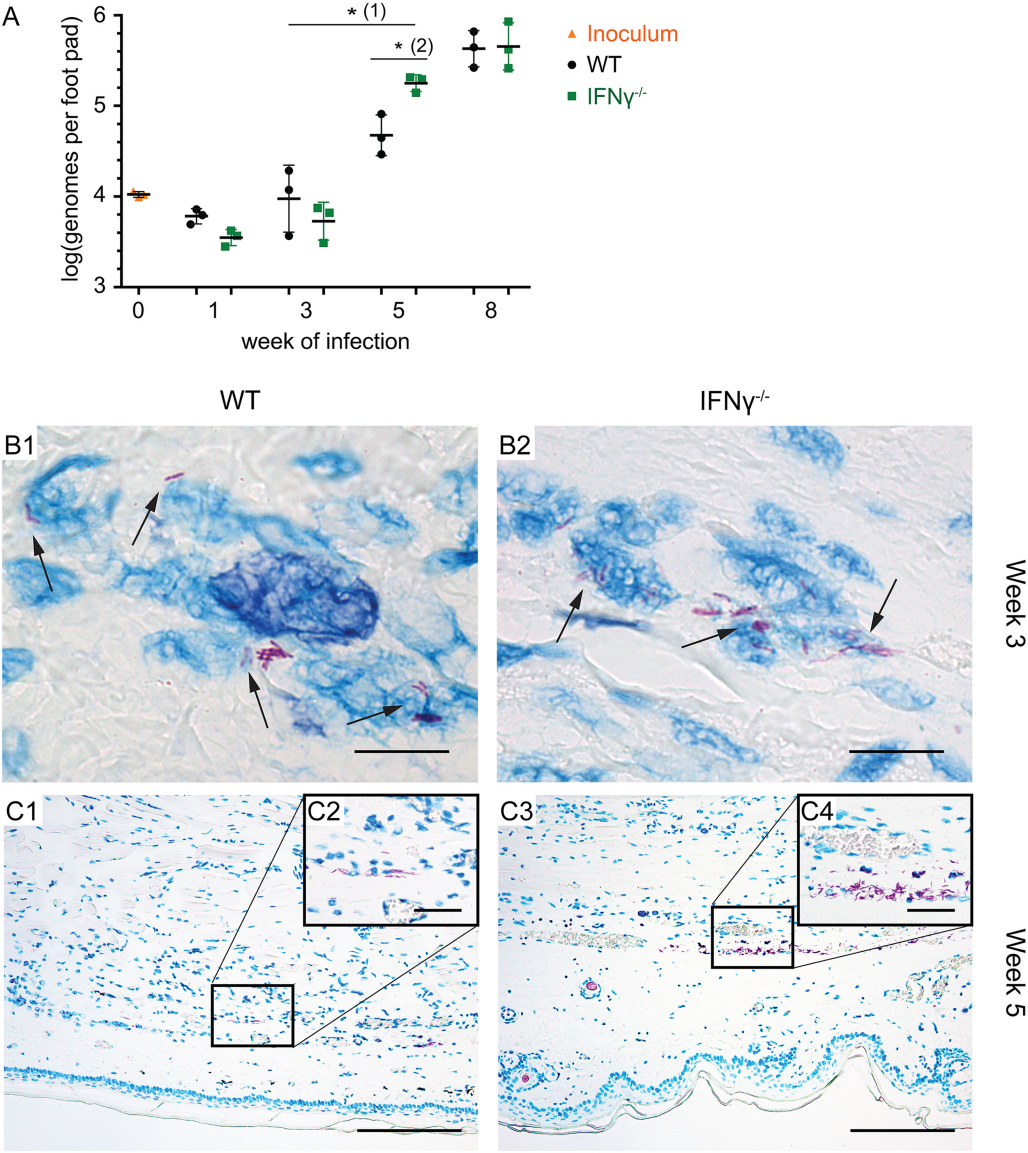
IFNγ-deficient mice have a significantly higher bacterial burden 5 weeks after infection. WT and IFNγ^-/-^ mice were infected with *M*. *ulcerans* and the bacterial load was determined by IS2404-specific qPCR (A). The distribution of AFB in the footpads was assessed by histopathological analysis at week 3 (B) and week 5 (C). (A) IFNγ^-/-^ mice showed a significantly stronger increase in the bacterial burden between week 3 and 5 (1) and had a significantly higher bacterial burden as compared to WT animals 5 weeks after infection with *M*. *ulcerans* (2). Values are displayed as mean, the error bars represent the S.D. (n = 3 per genotype). P values were calculated using non-parametric regression models according to the Brunner-Langer method. *, P ≤ 0.05. (B and C) 5 μm tissue sections of foot pads from representative WT (left) and IFNγ^-/-^ (right) mice stained with ZN for visualization of AFB after 3 (B) or 5 (C) weeks of infection. AFB were predominantly intracellular at week 3 (B1 and B2, black arrows) whereas a mix of intra- and extracellular bacilli was found after 5 weeks of infection (C). At week 5, more AFB were present in IFNγ^-/-^ foot pads (C2 and C4), no difference in the total immune cell infiltration between the two groups was observed (C1 and C3). Scale bars represent 8 μm (B1 and B2), 160 μm (C1 and C3) and 40 μm (C2 and C4).

Next, we assessed whether the more severe course of *M*. *ulcerans* infection in the IFNγ^-/-^ mice was associated with a higher bacterial burden in these animals. The bacterial load in footpads of three WT and three mutant mice was determined 1, 3, 5 and 8 weeks after infection by qPCR [23,34,35]. Strikingly, IFNγ^-/-^ mice showed a significantly higher increase in the bacterial load between week 3 and 5 (Fig 3A). Furthermore, we found that the bacterial load in the mice lacking IFNγ was significantly (3.5 fold) higher after 5 weeks of infection than in WT mice (Fig 3A), correlating with the strong foot pad swelling observed at this time point in only the mutant mice (Fig 1A and 1B). As for the foot pad thickness, the differences in the bacterial load had resolved 8 weeks after infection (Fig 3A).

To complement the qPCR results we stained tissue sections of whole foot pads with ZN to detect AFB. After 3 weeks of infection, only few AFB were found which were predominantly intracellular (Fig 3B1 and 3B2). As for the qPCR analysis (Fig 3A), no difference in the total number of AFB was observed between the two groups at this time point. However, a trend to less extracellular bacterial debris and more intact extracellular bacilli was observed for IFNγ^-/-^ foot pads at this time (S1 Fig).

In contrast, more AFB were detected in both IFNγ^-/-^ mice 5 weeks after infection (Fig 3C3 and 3C4), as compared to the two WT controls (Fig 3C1 and 3C2), which again corresponded with the results of the qPCR analysis (Fig 3A). At this time point, AFB were present as a mix of intra- and extracellular bacteria (Fig 3C). Interestingly, while the bacterial load was different for the two groups at this time point, no marked differences in the total cell infiltration was observed (Fig 3C). In line with the findings from the qPCR analysis (Fig 3A), the differences in the bacterial load had resolved 8 weeks after infection (S2 Fig).

### Lack of antibody responses against *M*. *ulcerans* after 5 and 8 weeks of infection

**Fig 4:**
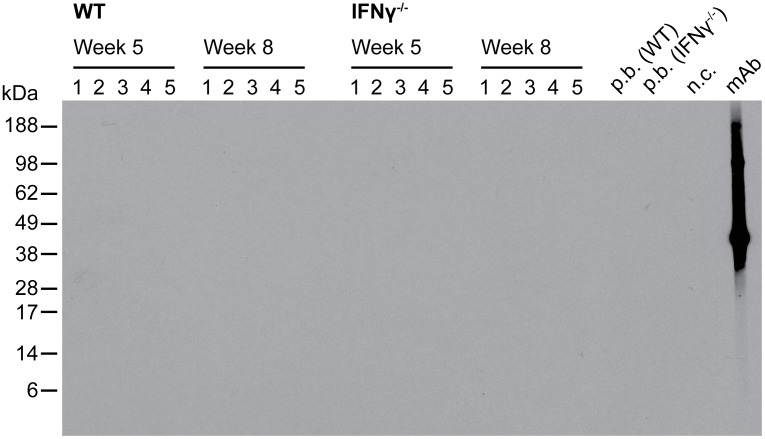
Absence of specific antibody responses against *M*. *ulcerans* in infected WT and IFNγ^-/-^ mice. Sera of WT and IFNγ^-/-^ mice were analyzed 5 and 8 weeks after infection for the presence of specific IgG antibody responses against *M*. *ulcerans* by Western blotting on *M*. *ulcerans* whole cell lysate. A monoclonal antibody specific for the *M*. *ulcerans* antigen MUL3720 served as positive control.

To evaluate whether the stronger increase in the bacterial load between weeks 3 and 5 in the IFNγ^-/-^ mice (Fig 3A) was caused by a diminished innate immune response as a result of lack of activating IFNγ or rather by reduced antibody-mediated immune responses against *M*. *ulcerans*, we tested the reactivity of sera of infected mice with *M*. *ulcerans* whole cell lysates by Western Blot analysis. A complete absence of specific antibodies was observed both for the five WT and five IFNγ^-/-^ mice after 5 and 8 weeks of infection (Fig 4). Together with the observed presence of less extracellular debris in IFNγ^-/-^ mice during the early phase of the infection (S1 Fig), this indicates that CMI is critical for host immunity against *M*. *ulcerans* infections.

## Discussion

Evidence for an early intra-macrophage growth phase of *M*. *ulcerans* has led to the suggestion that the immune effector mechanisms protecting against *M*. *ulcerans* infection are similar to those active against *M*. *tuberculosis* [41–43]. However, in contrast to this closely related pathogen, *M*. *ulcerans* has the capacity to produce the cytotoxic macrolide mycolactone, which eventually kills the host cells and causes the characteristic necrotizing pathology of BU [1,40]. In the case of *M*. *tuberculosis* infection, the host immune response involves cell-mediated immunity (CMI) accompanied by a delayed type hypersensitivity (DTH) reaction [44]. Similarly, several reports showed that CMI and DTH responses are frequently induced in BU patients [43,45–50].

If CMI is required for immunological defense against *M*. *ulcerans* infections, IFNγ which is produced primarily by T_H_1, but also by T_C_ and NK cells, is likely to play a critical role in this process by activating macrophages to kill intracellular bacteria at an early stage of infection. To test this hypothesis, we have used an experimental BU mouse model and compared the disease progression in WT and IFNγ^-/-^ mice during active infection with a highly virulent *M*. *ulcerans* strain recently isolated from the lesion of a BU patient [33]. Our study conclusively demonstrates a key role of IFNγ for early immune defense against *M*. *ulcerans* infection *in vivo*, as mice lacking this cytokine suffered from an accelerated and more severe pathology associated with a significantly higher bacterial burden after 5 weeks of infection. These results indicate that CMI and IFNγ-dependent activation of the bactericidal activity of macrophages helps to contain the infection during its largely intracellular early stages. Further support for this hypothesis came from our histopathological analysis, where a trend to lower levels of extracellular acid-fast debris was found in the IFNγ^-/-^ mice at the early intracellular stages of the infection.

Moreover, these findings are in line with the observation of *Torrado et al*. who have reported that IFNγ-dependent phagosome maturation and NO production are required to control the intracellular proliferation of *M*. *ulcerans in vitro* [32]. In the same report, it is described, that IFNγ-deficient mice show increased susceptibility only for mycolactone-negative or intermediate virulent, but not for highly virulent *M*. *ulcerans* strains [32]. However, these at first view contradictory results can be explained by the fact that the mice infected by *Torrado et al*. with a highly virulent *M*. *ulcerans* strain were only monitored over a period of 20 days post infection, a time frame that is too narrow to detect the differences between WT and IFNγ^-/-^ mice, as we did not observe them before 5 weeks of infection. In addition, different *M*. *ulcerans* strains differing in the geographic origin, the mycolactone variants produced and the pattern of genomic changes associated with evolutionary genome reduction [51] were used by the two groups.

In conclusion, our results indicate that the outcome of an infection with *M*. *ulcerans* may depend strongly on cellular immune defense mechanisms. IFNγ is likely to play an important role both as an element of innate immunity in the very early phase of host-pathogen interaction after inoculation and also in the subsequent development of protective adaptive cellular immune responses. Innate and adaptive immune defense mechanisms seem to be strong enough in the majority of exposed individuals living in BU endemic areas to protect them from developing clinical disease [13,14]. However, when the immune response of an individual is too weak to kill the intracellular bacteria, BU disease may develop. In line with this, HIV positive individuals are at higher risk for BU and AIDS-associated immunosuppression has a negative influence on the severity of BU [52–54]. In the case of an insufficient immune response, intracellular multiplication of the bacteria may take place and small accumulations of bacteria found as globus-like structures [30,55] may represent the origin for the formation of large clusters of mycolactone producing *M*. *ulcerans* bacteria. As a result of mycolactone-induced host cell apoptosis, necrotic areas are forming around the bacteria. Furthermore, in the advanced BU lesions viable leukocyte infiltrates are no longer found close to the infection foci in the necrotic subcutaneous tissue, indicating that the accumulation of mycolactone is preventing macrophages and other defense cells from reaching the now extracellular pathogens before they are killed. As a result, a chronic *M*. *ulcerans* infection may develop, leading to the formation of large BU lesions, often resulting in severe morbidity and disability and requiring long and costly hospitalization [40].

## Supporting Information

S1 Fig IFNγ-deficient mice show lower amounts of bacterial debris and higher numbers of intact extracellular bacteria 3 weeks after infection. Histologic analysis of foot pad sections from representative WT (left) and IFNγ^-/-^ (right) infected for 3 weeks of infection with *M*. *ulcerans*. Arrows indicate bacterial debris (left) or intact AFB (right). Scale bars, 8 μm.
(TIF)

S2 Fig No differences in the amount of AFB after 8 weeks of infection. Histologic sections of foot pads from representative WT (left) and IFNγ^-/-^ (right) mice infected for 8 weeks with *M*. *ulcerans* stained with ZN for AFB visualization. Scale bars, 30 μm.
(TIF)
