# Supplementary figures and images for: Interferon-γ Is a Crucial Activator of Early Host Immune Defense against Mycobacterium ulcerans Infection in Mice
Source: PLoS Negl Trop Dis. 2016 Feb 10;10(2):e0004450. doi: 10.1371/journal.pntd.0004450 (PMC4749296; doi:10.1371/journal.pntd.0004450)

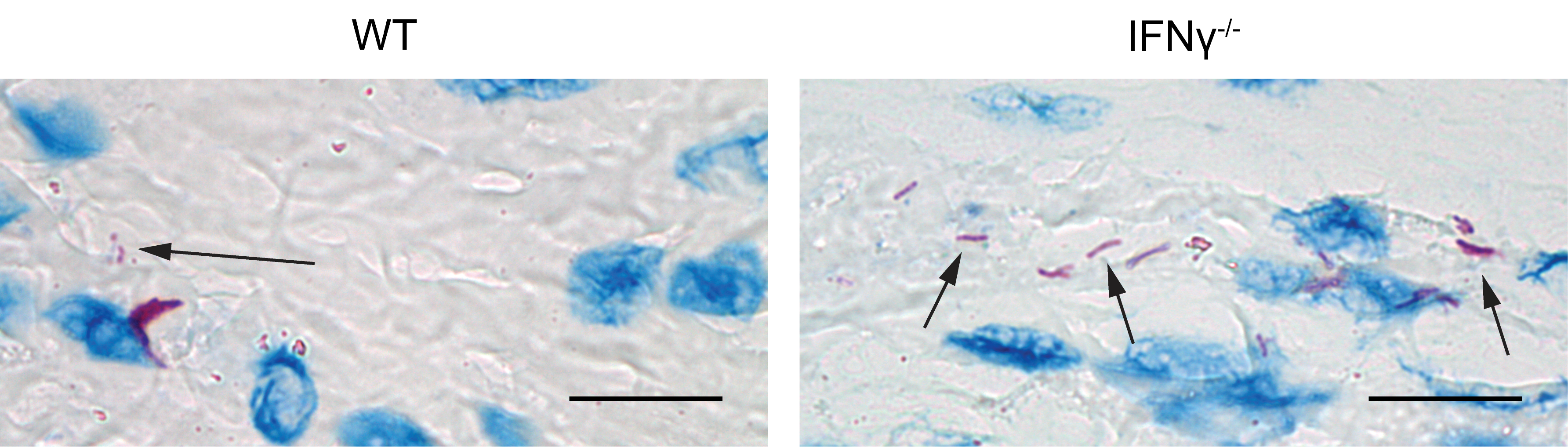

Supplement: S1 Fig — Histologic analysis of foot pad sections from representative WT (left) and IFNγ-/- (right) infected for 3 weeks of infection with M. ulcerans. Arrows indicate bacterial debris (left) or intact AFB (right). Scale bars, 8 μm. (TIF) [file pntd.0004450.s001.tif]

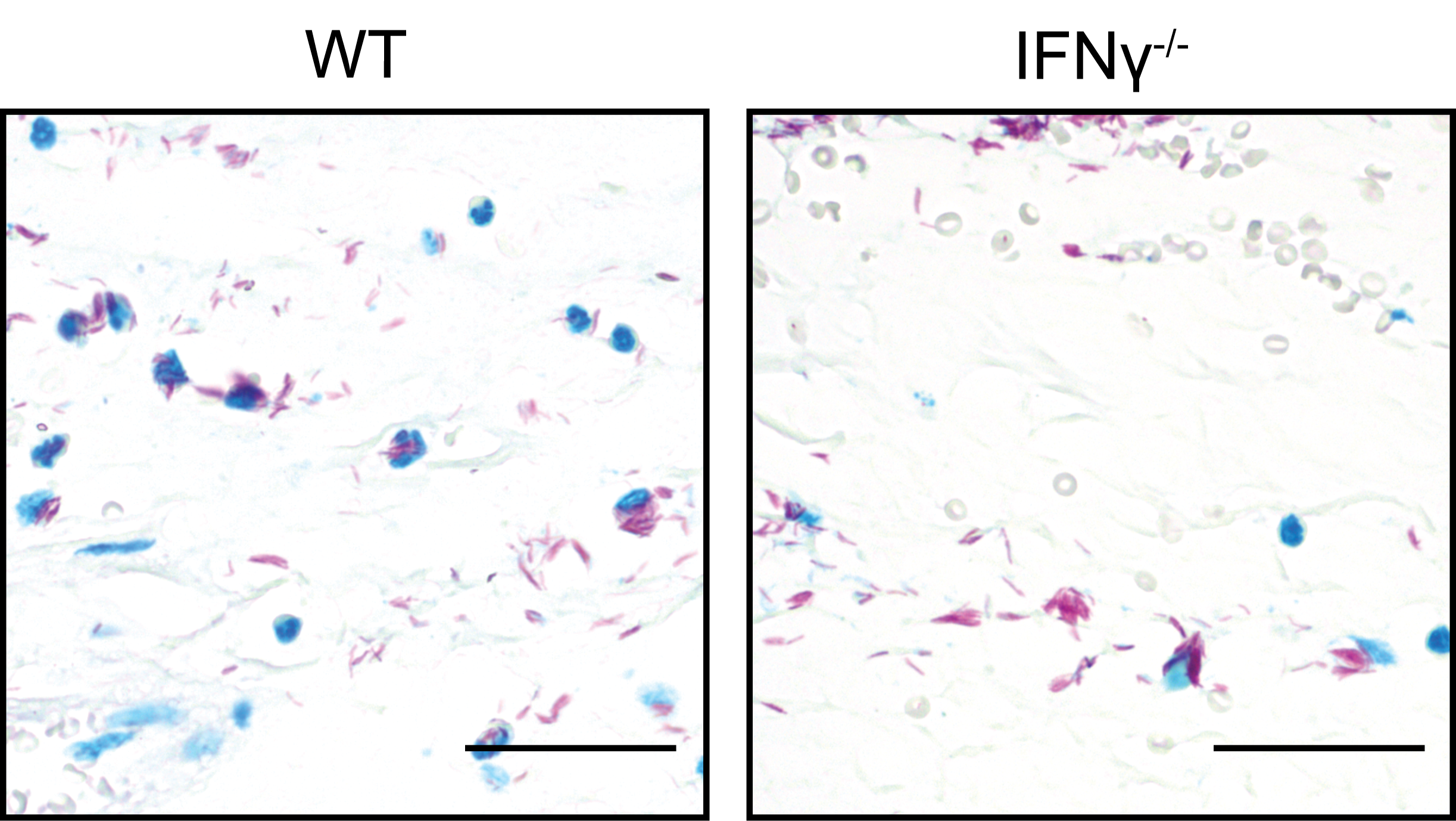

Supplement: S2 Fig — Histologic sections of foot pads from representative WT (left) and IFNγ-/- (right) mice infected for 8 weeks with M. ulcerans stained with ZN for AFB visualization. Scale bars, 30 μm. (TIF) [file pntd.0004450.s002.tif]
